# Biological Susceptibility of Patients with Schizophrenia to Metabolic Syndrome: A Review

**DOI:** 10.31083/AP39896

**Published:** 2025-04-22

**Authors:** Jun Ma, Zhengyuan Huang, Jing Chen, Gaohua Wang

**Affiliations:** ^1^Department of Psychiatry, Renmin Hospital of Wuhan University, 430060 Wuhan, Hubei, China; ^2^College of Life Science and Technology, Wuhan Polytechnic University, 430023 Wuhan, Hubei, China; ^3^Taikang Center for Life and Medical Sciences, Wuhan University, 430071 Wuhan, Hubei, China; ^4^Institute of Neuropsychiatry, Renmin Hospital of Wuhan University, 430060 Wuhan, Hubei, China

**Keywords:** schizophrenia, metabolic disorder, genetics, lifestyle, antipsychotic drug

## Abstract

Schizophrenia (SCZ) is a debilitating, chronic mental disorder with an elusive etiology that significantly impacts the life expectancy of affected individuals. Metabolic syndrome (MetS) is a condition characterized by a combination of factors that increase the risk of cardiovascular diseases. MetS is more prevalent in individuals with SCZ and is a major factor that contributes to their reduced lifespan. This review scrutinizes the biological factors that predispose patients with SCZ to MetS, among which, genetic predisposition, dietary and lifestyle modifications, and the use of antipsychotic drugs (APs) play a significant role. The metabolic side effects of APs have been well studied. While studies have shed light on potential interventions to manage MetS in patients with SCZ, identifying precise biological targets to treat SCZ remains challenging. Therefore, further studies are warranted to enhance our comprehension of the intricate mechanisms underlying the susceptibility of patients with SCZ to MetS. These studies will be crucial in developing effective, targeted therapeutic strategies to treat MetS in this vulnerable population.

## Main Points

(1) Patients with schizophrenia are prone to metabolic syndrome via intricate underlying mechanisms.

(2) The interplay of genetic susceptibility, unhealthy lifestyle, and antipsychotic drugs contributes to the onset of metabolic syndrome.

(3) Antipsychotic drugs contribute to the development of metabolic syndrome through multiple pathways, including pharmacogenetics, neuroendocrine hormonal regulation, and inflammatory processes.

(4) Specific targets for intervention in metabolic syndrome associated with schizophrenia are unclear and warrant further investigation.

## 1. Introduction

Schizophrenia (SCZ) is a severely debilitating, chronic mental disorder characterized by an unknown etiology and signs and symptoms including hallucinations, delusions, disorganized speech, and disturbed behavior [1]. SCZ poses a serious threat to the physical health of patients, resulting in a reduction in life expectancy by approximately 15 years [2, 3]. Metabolic syndrome (MetS) is a combination of cardiovascular disease risk factors such as abdominal obesity, hypertension, dyslipidemia, and insulin resistance [4, 5]. It is a major health hazard in modern society that is only second to infectious diseases [6]. The global prevalence of MetS is increasing rapidly and affecting children and adolescents as well [7, 8]. MetS is particularly prevalent in patients with SCZ. It is estimated that 37%–67% of patients with SCZ are comorbid with MetS, a rate that is 2–3 times higher than in the general population [9, 10]. High susceptibility to MetS is one of the most common causes of premature death in patients with SCZ [11, 12]. Furthermore, MetS leads to poor cognitive function in patients with SCZ [13, 14] and worsens the already severe damage to the central nervous system (CNS) [15]. Therefore, there is an urgent need to identify novel approaches to prevent the development and progression of MetS in the SCZ population.

Research focusing on the mechanisms of MetS in comorbid SCZ has become increasingly critical due to its significant impact on the lifespan and long-term prognosis of patients. This review critically summarizes previous reports of SCZ in combination with the mechanisms of MetS published over the past decade, highlighting key advances in research and identifying persistent gaps and limitations.

## 2. Genetic Overlap between SCZ and MetS

Research findings indicate that SCZ is associated with metabolic dysregulation, which is independent of the use of antipsychotic drugs (AP). Notably, disruptions in glucose metabolism and insulin resistance are observed at disease onset even without the influence of AP drugs [16, 17]. Individuals experiencing their first episode of SCZ are at a higher risk of exhibiting at least one component of MetS compared with the general population [18]. This risk is further exacerbated by a significant increase in the overall prevalence of MetS among these patients [19], prompting investigations into the genetic and hereditary mechanisms underlying this debilitating comorbidity [20].

Genetic analyses, such as linkage disequilibrium score regression, have led to the identification of genetic correlations between SCZ and the various components of MetS across East-Asian and European populations [21]. These findings are supported by a large-scale genome-wide study that includes a joint analysis with the Genotype-Tissue Expression project [22]. This project has identified 22 genomic loci shared between SCZ and MetS, with 49 overlapping genotypes in tissue expression. Propionyl-CoA carboxylase and Potassium Channel Tetramerization Domain Containing 13 (*KCTD13*) genes, specifically, have been implicated as potential genetic mediators of this association [22].

Despite the genetic overlap, the expected reciprocal increase in the susceptibility to and prevalence of SCZ among patients with MetS has not been observed. This asymmetry in genetic predisposition suggests that relying solely on static genetic factors to identify intervention targets for MetS in the SCZ population could be limited. Therefore, there is a pressing need for further research related to dynamic and modifiable factors to develop effective strategies to manage MetS in individuals with SCZ.

## 3. Adverse Lifestyle Influences the Development of MetS in Patients with SCZ

An unhealthy lifestyle significantly contributes to the increased susceptibility of patients with SCZ to MetS [23, 24]. Patients with SCZ are less physically active compared with healthy individuals, and a significant number of patients engage in minimal or no aerobic exercise [25]. Furthermore, up to 42% of these patients do not participate in any form of vigorous physical activity on a weekly basis [26]. Additionally, they are more inclined toward the consumption of carbonated beverages rather than following a balanced diet of fruits and vegetables [26, 27]. Unhealthy behaviors such as smoking, excessive alcohol intake, and sleep deprivation are more common among patients with SCZ [28, 29, 30]. These habits not only profoundly exacerbate cardiovascular health issues but also increase the risk of developing MetS [31]. The presence of adverse symptoms and cognitive deficits in patients with SCZ can further exacerbate the development of unhealthy lifestyle habits, in turn contributing to the onset of MetS [32, 33].

While the precise impact of lifestyle on MetS is challenging to determine due to its varied and intricate manifestations, published studies strongly indicate that addressing unhealthy lifestyle habits and advocating a wholesome diet are key strategies to mitigate MetS.

## 4. Mechanisms of AP–Induced MetS

Although APs are an indispensable therapeutic intervention in the management of SCZ, drugs such as olanzapine and clozapine are responsible for inducing MetS in these patients [34, 35]. It has been reported that patients with SCZ exposed to APs may exhibit a prevalence of MetS of up to 63% [36]. Even APs such as aripiprazole and ziprasidone, which are known for their favorable metabolic profile, are associated with an increased risk of MetS to some extent [34, 37]. Although a large meta-analysis has confirmed varying degrees of the risk of MetS associated with different atypical APs, the efficacy and safety of low-metabolic-risk medications do not always align with expectations during prescription and management [38]. Consequently, AP drugs such as olanzapine and clozapine, which are associated with high metabolic risks, are widely prescribed despite their effectiveness [38, 39, 40], suggesting that metabolic disturbances may be inevitable in patients prescribed these drugs. Based on these findings, there has been an increased research focus to elucidate the mechanisms by which APs trigger MetS. This line of inquiry is crucial for developing strategies to mitigate the metabolic risks associated with AP use in patients with SCZ.

### 4.1 Gene Polymorphisms Mediate AP-Induced MetS

Significant progress in pharmacogenomics over the past decade has intensified the investigation into the role of specific gene polymorphisms in the etiology of druginduced MetS, making it a highly promising area of research [41]. A summary of pertinent findings is presented in Table 1 (Ref. [42, 43, 44, 45, 46, 47, 48, 49, 50, 51]).

**Table 1.** **Findings from 10 studies of MetS risk gene polymorphisms in SCZ patients after antipsychotic exposure**.

Reference	Study setting	Participant characteristics	Factors (exposure)	Gene: Loci	MetS risk
		Sample size; Diagnosis			
		(MetS/non-MetS); age			
Yang, L. *et al*. 2015 [42]	China	621; 260/361; 44.42 ± 10.76	Clozapine	*SREBF2*: rs1052717	OR = 1.67
				*SREBF2*: rs2267443	OR = 1.81
Yang, L. *et al*. 2016 [43]	China	722; 291/431; 43.73 ± 10.95	Clozapine, Olanzapine, Risperidone	*SREBF1*: rs11654081-T	OR = 2.56
Zhou, W. *et al*. 2022 [44]	China	990; 444/546; 48.7 ± 15.0	Open-ended	*leptin/PPAR* gene sets: NA.	MetS↑
Boiko, A. S. *et al*. 2022 [45]	Russian	517; 139/378; 44 (34, 54)	Open-ended	*FTO* gene: NA.	Not MetS but central besity↑
Roffeei, S. N. *et al*. 2014 [46]	Malaysia	206; 123/83; 39.90 ± 11.29	Open-ended	*FTO* gene: rs9939609	OR = 1.73
Boiko, A. S. *et al*. 2021 [47]	Russian	517; 139/378; 44.19 ± 11.51	Open-ended	*LEP*: rs3828942	OR = 2.06
				*INSIG2*: rs17047718	OR = 1.40
Kim, E. Y. *et al*. 2016 [48]	South Korea	391; NA/NA; 38.51 ± 11.62	Open-ended	*DBP*: rs2838543-C	Male but female
					OR = 1.719
Fattakhov, N. *et al*. 2018 [49]	Russian	345; 155/190; 46.54 ± 13.08	Open-ended	*NOS3*: rs2070744(T-786C)	OR = 0.59
				*NOS3*: rs2070744(T-786TT)	OR = 0.45
Mednova, I. A. *et al*. 2024 [50]	Russian	489; 131/358; 44 (34, 54)	Open-ended	*NOS1AP*: rs10494366-T	OR = 1.60
				*NOS1AP*: rs12143842-C	OR = 1.75
Pinto, J. A. F. *et al*. 2018 [51]	Brazil	72; 34/38; about 43 years	Clozapine	*DRD2*: -141C- Ins C	Not MetS but low-HDL
					OR = 19.8

Age: mean age in the MetS group; Open-ended: no restriction on types of antipsychotics; SREBF2, sterol regulatory elements bind transcription factor 2; SREBF1, sterol regulatory elements bind transcription factor 1; PPAR, peroxisome proliferator-activated receptors; FTO, fat mass and obesity associated gene; LEP, leptin gene; INSIG2, Insulin-inducible gene 2; DBP, D site of albumin promoter binding protein; NOS3, endothelial nitric oxide; NOS1AP, Nitric oxide synthase 1 articulating protein; DRD2, dopamine receptors of D2; NA, not applicable or not available; OR, odds ratio; HDL, high density lipoprotein; ↑, elevated levels; Mets, metabolic syndrome; SCZ, schizophrenia.

Sterol regulatory element binding protein (SREBP) transcription factors, classified as SREBP1 and SREBP2, encoded by the sterol regulatory element binding transcription factor 1 (*SREBF1*) and *SREBF2* genes, located on chromosomes 17p11.2 and 22q13.2, respectively, are key factors regulating fatty acid and cholesterol biosynthesis [52]. Yang* et al*. [42, 43] studied the effect of SREBF on MetS in patients with SCZ. Initially, they reported that single nucleotide polymorphisms in *SREBF2* could predict MetS in patients treated with clozapine [42]. In-depth evaluation of the classes of exposure to APs revealed that the rs11654081-T allele of *SREBF1* was also associated with an elevated risk of MetS [43].

Regulatory genes for neuroendocrine hormones and the “fat mass and obesity associated” (*FTO*) gene have garnered significant scholarly interest. A recent multiomics analysis across multiple centers has identified rare functional variants in leptin and peroxisome proliferator–activated receptors, which are differentially expressed in AP-induced MetS than in the non-MetS group [44]. Boiko* et al*. [45] conducted a detailed analysis of polymorphisms in insulin-inducible gene 2 (*INSIG2*), growth hormone-releasing peptide (GHRL), leptin (LEP), and leptin receptor (*LEPR*) genes, revealing significant associations between specific genotypes and the development of MetS. Additionally, the rs9939609 variant of the *FTO* gene was identified as a risk factor for MetS in Malaysian patients and for the body mass index (BMI) in Russian patients [46, 47].

Studies related to polymorphisms in genes associated with protein promoters, such as the albumin promoter binding protein (DBP) and endothelial nitric oxide synthase (NOS3), have also been noteworthy. Kim* et al*. [48] have reported associations between the C allele of rs3848543 of DBP and the severity of MetS in male Korean patients with SCZ. Similarly, T-786C polymorphism (rs2070744) in the promoter region of *NOS3* has been reported to be strongly linked to an increased risk of MetS in Russian patients with SCZ [49]. Further study related to nitric oxide synthase 1 articulating protein (*NOS1AP*), which regulates NOS3, has confirmed the associations between 2 nucleotide polymorphisms (rs12143842 and rs10494366) and the risk of MetS in Russian patients [50].

Genes that modulate dopamine signaling, affecting eating behavior through reward pathways, have been implicated in obesity and dyslipidemia [53]. Pinto* et al*. [51] have established a correlation between the dopamine gene family and clozapine-induced MetS, particularly noting the -141C polymorphism in the dopamine receptors of D2 (*DRD2*) gene to be associated with low high-density lipoprotein (HDL) levels.

Epigenetic factors, such as methylation of key genes due to oxidative stress, may also contribute to the development of MetS [54]. Studies [44, 55] have reported higher methylation levels of *CDH22* and *ABCG1* genes in the MetS subgroup versus that in the non-MetS subgroup.

While research into the genetic and epigenetic mechanisms of AP-induced MetS is both extensive and insightful, the findings vary considerably. This variation presents challenges in identifying universally effective targets to prevent or treat MetS. However, these studies do provide valuable insights into the genetic mechanisms by which APs may induce MetS, suggesting a significant regulatory role via the interaction between genetic variants and metabolic profiles in patients with SCZ.

### 4.2 Effects of APs on MetS-Related Neuroendocrinology

Neuroendocrine involvement in mediating MetS is another research hotspot [56, 57]. The hypothalamic–pituitary–adrenal (HPA) axis, hypothalamic–pituitary–gonadal (HPG) axis, growth hormone (GH)-insulin-like growth factor-1 (IGF-1) (GH-IGF-1) axis, and hypothalamic–pituitary–thyroid (HPT) axis have been studied to comprehend their role in AP-induced MetS. The main results of relevant studies are summarized in Table 2 (Ref. [56, 58, 59, 60, 61, 62, 63, 64, 65, 66, 67, 68, 69, 70, 71]).

**Table 2.** **Results of 15 studies on MetS-related neuroendocrine changes in SCZ patients exposed to antipsychotics**.

Reference	Research Design	Participant characteristics	Factors (exposure)	Neuroendocrine axis: hormone	Outcome measure
		Sample size; Diagnosis			
		(MetS/non-MetS); age			
van den Heuvel, L. L. *et al*. 2022 [58]	South Africa, Longitudinal	37; NA/NA; 30.1 ± 6.9	Open-ended	HPA: HCC	HCC↑
Vuksan-Ćusa, B. *et al*. 2014 [59]	Croatia, Case-Control	123; 43/80; 42.8 ± 13.7	SGA	HPA: DHEAS, cortisol	Not MetS but DBP, cortisol/DHEAS↑
Boiko, A. S. *et al*. 2020 [60]	Russian, Case-Control	110; 42/68; 42.42 ± 11.17	Open-ended	HPA: DHEAS, cortisol	DHEAS↓ in co-MetS females
Zhang, H. *et al*. 2023 [61]	China, Case-Control	93; 14/79; 49 (37, 54)	Open-ended	HPG: Prolactin, Estradiol, Testosterone	Prolactin↑ in older MetS females
Wu, T. H. *et al*. 2020 [62]	China, Case-Control	151; 41/110; 41.4 ± 10.6	Olanzapine	GU- IGF-1: AG, DAG	AG/DAG↑
Demirel, A. *et al*. 2014 [63]	Turkey, Case-Control	50; 21/29; 36.46 ± 11.20	SGA	GU- IGF-1: IGF-1	Not MetS but HDL,
					IGF-1↑
Ünal, K. *et al*. 2018 [64]	Turkey, Case-Control	55; 11/44; 36.01 ± 10.10	Open-ended	NA: Nesfatin-1	Nesfatin-1↑
Kornetova, E. G. *et al*. 2020 [65]	Russian, Cross-Sectional	156; 56/100; 45.5 (35.5, 54)	Open-ended	HPT: TSH, FT_3_, FT_4_	FT_3_↑, FT_4_↑
Kalinowska, S. *et al*. 2019 [66]	Poland, Longitudinal	106; 64/42; 41.89 ± 9.70	Open-ended	HPT: TSH, FT_3_, FT_4_	Hypothyroidism impairing FBG not MetS.
Boiko, A. S. *et al*. 2022 [56]	Russian, Case-Control	195; 95/100; 44.5 (33.75, 52.25)	Open-ended	NA: leptin, ghrelin	Leptin↑, ghrelin↓
Mednova, I. A. *et al*. 2020 [67]	Russian, Case-Control	110; 46/64; 39.5 (30, 51)	Open-ended	NA: leptin, adiponectin	Leptin↑, leptin/adiponectin↑
Chen, V. C. H. *et al*. 2018 [68]	China, Case-Control	262; 87/175; 42.5 ± 10.6	Clozapine or Olanzapine	NA: leptin, adiponectin	Leptin↑, adiponectin↓, leptin/adiponectin↑
Tay, Y. H. and Lee, J. 2019 [69]	Singapore, Case-Control	81; NA/NA; 36.19 ± 7.65	Open-ended	NA: adiponectin	Adiponectin↓
Chen, P. Y. *et al*. 2019 [70]	China, Case-Control	157; 49/108; NA	Clozapine and	NA: orexin-A	Orexin-A↓
			non-Clozapine		
Chen, P. Y. *et al*. 2022 [71]	China, Case-Control	201; NA/NA; 38.2 ± 12.7 or 42.3 ± 9.8	Clozapine and Aripiprazole	NA: orexin-A	Orexin-A↓

Age: mean age in the MetS group; Open-ended: no restriction on types of antipsychotics; NA, not applicable or not available; HPA, hypothalamus-pituitary-adrenal; HPG, hypothalamus-pituitary-gonadal; GU- IGF-1, growth hormone-insulin-like growth factor-1; HPT, hypothalamus-pituitary-thyroid; HCC, hair cortisol concentration; DHEAS, dehydroepiandrosterone; DBP, diastolic blood pressure; AG, acylated growth hormone-releasing peptide; DAG, deacylated growth hormone-releasing peptide; IGF-1, insulin-like growth factor-1; HDL, high-density lipoprotein; TSH, thyroid-stimulating hormone; FT_3_, free triiodothyronine; FT_4_, free tetraiodothyronine; FBG, fasting blood glucose; SGA, Second-generation antipsychotics; ↑, elevated levels; ↓, decreased levels.

Glucocorticoids, including cortisol and dehydroepiandrosterone sulfate (DHEAS), are the main products secreted by the HPA axis. Its main functions are to promote hepatic gluconeogenesis, inhibit glucose uptake, promote lipolysis, and inhibit insulin secretion, eventually leading to insulin resistance and metabolic disorders [72]. Long-term cortisol secretion and the cortisol-to-DHEAS ratio are elevated in patients with MetS after exposure to APs [58, 59], whereas DHEAS levels are reduced [60]. DHEAS supplementation in patients with SCZ can lead to a significant worsening of metabolic markers such as BMI, waist circumference, and fasting glucose levels [73]. A case-control study focusing on the HPG axis examined the relationship between prolactin, estradiol, and testosterone levels and MetS. Elevated serum prolactin levels were found to be strongly associated with the risk of MetS in patients with SCZ, especially in women over 50 years of age [61].

Recent studies have explored the effects of GH-IGF-1 axis–related hormones on the development of MetS in patients with SCZ. Growth hormone–releasing peptide regulates GH release from the pituitary gland. It contains the acylated growth hormone–releasing peptide (AG) and deacylated growth hormone–releasing peptide (DAG) forms, both of which mediate glucose homeostasis and lipid metabolism. AG promotes the development of MetS and type 2 diabetes mellitus, whereas DAG has the opposite effect [74, 75]. Patients with SCZ having MetS show a higher AG-to-DAG ratio, which also mediated higher MetS scores [62]. IGF-1 is lipolytic. Its production is dependent on GH, and it has a negative feedback regulation on GH secretion [76]. IGF-1 levels in the SCZ population are positively correlated with HDL levels [63]. Researchers have also found that the level of Nesfatin-1, which has a physiological function similar to that of DAG, is also significantly elevated in patients with SCZ having MetS [64]. Collectively, these findings indicate that an imbalance of the GH-IGF-1 axis plays a role in the development of MetS in patients with SCZ.

The HPT axis is another well-known neuroendocrine system that influences metabolic functions and exerts a potent effect on systemic and hepatic lipid metabolism in mammals [77]. In Western Siberia, free triiodothyronine and thyroxine levels were higher in patients with SCZ having comorbid MetS versus those in patients without comorbid MetS [65]. In the setting of baseline hypothyroidism (thyrotropin ≥4.50 µIU/mL), poor metabolic status has been reported to be more common in patients with SCZ at future follow-up [66]. This finding indicates that maintaining normal thyroid function can be helpful in maintaining good metabolic levels.

Common adipokines have attracted considerable research attention. Leptin and lipocalin are peptide or protein-like hormones secreted by adipose tissues. These hormones may play a role in the development of MetS [78] but they certainly do affect the development of MetS in patients with SCZ [56, 67]. Leptin levels and the leptin-to-lipocalin ratio are elevated in patients with SCZ having MetS [67, 68], whereas lipocalin levels are reduced [56, 67, 68, 69]. Moreover, serum lipocalin levels are known to decrease as the number of MetS components increases [69]. Although there is disagreement among these studies about which parameter is more recognized as the preferred marker of comorbid MetS in patients with SCZ [67, 68], they are all in considerable agreement with respect to the importance of leptin and lipocalin in the development of MetS.

Orexin-A is secreted by orexin neurons located in the lateral hypothalamic region. It increases lipolysis and insulin sensitivity and exerts protective effects related to metabolism [79, 80]. The orexin-A signaling system plays an important role in AP drug–associated metabolic dysregulation. Exposure to APs has can increase orexin-A levels in patients with SCZ. Furthermore, aripiprazole was found to modulate orexin A levels more than clozapine [70, 71]. This finding implies that higher modulation of orexin-A can help suppress metabolic abnormalities. Unfortunately, these researchers did not conduct prospective studies to determine the role of orexin-A in inhibiting the long-term development of MetS. Lastly, the levels of the most prominent anorectic neurotrophic factor in the CNS [81], brain-derived neurotrophic factor, are reported to be reduced in patients with chronic SCZ having MetS [82].

APs exert a range of effects on neurohormones associated with metabolism and affect multiple neuroendocrine axes and their corresponding target organs. The effects of APs on these neurohormonal factors in conjunction with the existing interactions among them may collectively form a mechanism via which APs contribute to the development of MetS.

### 4.3 Effect of APs on MetS-Related Immune Dysregulation

Immune dysregulation is being increasingly recognized as a significant factor responsible for both metabolic diseases and SCZ [83, 84]. Patients with SCZ often display signs of metabolic-inflammatory imbalance even during initial treatment stages [85]. Studies have consistently reported elevated levels of various inflammatory markers, such as white blood cells, C-reactive proteins, complement components, cytokines, and other inflammatory macromolecules, in patients with SCZ having MetS, especially after exposure to APs [86, 87, 88, 89, 90, 91]. The kynurenine metabolic pathway (KP) is closely linked to inflammatory diseases and plays a crucial role in the pathogenesis of SCZ [92, 93]. It is a logical focal point to understand the association between SCZ and MetS [94]. The interaction of SCZ with the KP contributes to the susceptibility to MetS [95]. Furthermore, prolonged exposure to APs can increase the levels of inflammatory markers, thereby exacerbating the risk of developing MetS [85, 96].

Sirtuin 1 (SIRT1), a downstream metabolite of the KP and a NAD^+^-dependent protein deacetylase, promotes lipid and glucose metabolism [97]. Studies have indicated that patients with SCZ having MetS tend to have lower plasma SIRT1 levels versus those without MetS or healthy individuals [90].

Although the exact mechanism of the interplay between SCZ, immune dysregulation, and MetS remains unclear, these factors are interconnected. Immune dysregulation may be a key factor in both the intrinsic development of MetS in patients with SCZ and the exacerbation of SCZ by APs. Therefore, targeting immune dysregulation could be a promising strategy for preventing and/or treating MetS in patients with SCZ.

### 4.4 Gut Microbiota Mediates MetS in Patients with SCZ

The gut microbiota is being increasingly recognized as a potential contributor to the development of neurological disorders including SCZ [98]. An imbalance in the gut microbiota due to the overgrowth of harmful bacteria and suppression of beneficial bacteria is closely linked to MetS [99]. Accordingly, researchers have been investigating the role of the gut microbiota in stabilized patients with SCZ who are prone to MetS, as it may serve as a bridge between these 2 conditions [100]. Factors such as the use of APs can mediate interactions between the gut microbiota and host metabolism [101, 102].

Several studies have delved deep into examining whether APs might exacerbate MetS by disrupting the composition of the gut microbiota [103, 104, 105]. A case-control study reported that patients with SCZ having MetS exhibited a notable decrease in bacterial α-diversity, significant variations in β-diversity, and shifts in bacterial genera that are associated with features of MetS compared with that in healthy individuals [103]. Further analysis showed distinct differences in the diversity, composition, and abundance of fecal microbiota between SCZ patients with and without MetS [104], and showed potential correlation with the severity of MetS in patients with SCZ [105].

Numerous studies have highlighted the potential role of the gut microbiota in AP induced MetS [102, 106]. However, several confounding factors, such as age and the type of APs and also the neglect of patients with SCZ who have MetS prior to treatment with AP, can complicate the direct attribution of the development of MetS and progression to AP-induced dysbiosis of the gut microbiota [106].

## 5. Conclusions

The susceptibility of patients with SCZ to MetS results from the complex interplay between intrinsic factors such as genetics, gut microbial disorders, and immune inflammation as well as extrinsic factors such as exposure to APs, dietary patterns, and lifestyle choices. Despite the relative stability of genes, the genes and loci associated with the development of MetS in SCZ are heterogeneous and unclear. The genes and loci identified in the context of drug-naïve individuals are rarely reconfirmed after exposure to APs, suggesting that these drugs may not contribute to the risk of MetS through genetic pathways that overlap with MetS. Furthermore, the genetic overlap between SCZ and MetS only explains the susceptibility of patients with SCZ to MetS, whereas only a few reports on patients with MetS being equally susceptible to SCZ have been published. More studies have reported gene-specific polymorphisms in AP-treated patients compared with investigations related to genetic overlap in drug-naïve individuals, suggesting that APs may confer a greater genetic risk of MetS. Moreover, the extensive disruption of neuroendocrine pathways and gene methylation by APs introduces further uncertainty in identifying therapeutic targets.

Despite the numerous uncertainties discussed in this review, the findings collectively offer valuable insights for future research to identify appropriate therapeutic targets to treat MetS. Therefore, a comprehensive understanding of the interplay between pharmacogenetics, immunoinflammation, neuroendocrinology, gut microbiota, and the pathways leading to MetS is essential. This could be achieved by designing robust clinical studies with larger cohorts to validate potential therapeutic targets. The findings will be instrumental in developing targeted molecular therapies for the management of MetS. In the interim, optimizing the use of APs, promoting a healthy diet, providing lifestyle education, and implementing interventions for glycolipid metabolism may continue to be effective strategies to manage MetS in patients with SCZ.
